# Artificial intelligence learning environments and educational psychology (2016–2025): a systematic bibliometric synthesis and review

**DOI:** 10.3389/fpsyg.2026.1831006

**Published:** 2026-08-06

**Authors:** Shujin Zhong, Jack D. Simons

**Affiliations:** 1 Department of Teaching, Learning and Curriculum, Silverfield College of Education and Human Services, University of North Florida, Jacksonville, FL, United States; 2 Department of Counseling, School of Social and Behavioral Sciences, Mercy University, Dobbs Ferry, NY, United States; 3 Department of Psychology, Faculty of Education, Charles University, Prague, Czechia

**Keywords:** artificial intelligence in education (AIEd), bibliometric review, educational psychology, generative AI, identity, metacognition, self-regulated learning

## Abstract

The rapid integration of Generative Artificial Intelligence (GenAI) and Large Language Models (LLMs) into educational environments has fundamentally transformed instructional design building upon earlier AI research in educational psychology. However, there is growing concern that computational innovation is outstripping pedagogical and psychological coherence. To evaluate the intersection of AI learning environments and educational psychology, this study provides a systematic bibliometric synthesis of peer-reviewed scholarship published between 2016 and 2025. Following PRISMA 2020 guidelines, we employed a dualpronged approach, applying multiple descriptive and analytical modeling techniques to analyze a synthetic corpus of 34 systematic review papers and an empirical corpus of 1,376 original research articles. Longitudinal results reveal an unprecedented “generative turn” and a shift in methodological maturity, moving from exploratory computational modeling to rigorous experimental and quantitative validation. Despite these advancements, our analysis identifies a persistent “theoretical gap.” While the synthetic literature advocates for deep psychological grounding, empirical studies remain overwhelmingly anchored in surface-level motivational outcomes, failing to adequately operationalize complex constructs such as metacognition, epistemic beliefs, and self-regulated learning (SRL), just to name a few. We conclude that for AI to foster effective, equitable instruction and learning, it must transcend being a mere content delivery mechanism and instead function as a “psychological catalyst.” By prioritizing human-centric architectures, cognitive scaffolding, identity, and critical AI literacy, this synthesis provides a structural roadmap for stakeholders to align rapid technological strides with the foundational science of learning.

## 1 Introduction

The rapid integration of Artificial Intelligence (AI) into educational design is fundamentally transforming instruction, assessment, and learning, including self-regulated learning, which has been studied most notably by Paul Pintrich (Schunk, 2005). More recently, we see the development of AI software coming largely from China (Liao, 2024). In addition, AI educational tools are becoming more personalized (McLaren et al., 2011; Schiaffino et al., 2008; VanLehn, 2011). For some, it is an exciting time, and for others it is a worrisome time, yet, no doubt we are now extending earlier AI research for computer use today in education (Atkinson, 1968; Atkinson and Wilson, 1969), as well as tutoring (Graesser et al., 1999; Psotka and Mutter, 1988), and even theory development (Collins, 1989). It is an unprecedented moment, but research on and advancements in AI in education are not coming about without strain and caution for some.

While computational capabilities have advanced exponentially, particularly with the recent “generative turn” and the rise of Large Language Models (LLMs), there is concern that technological innovation is outstripping pedagogical and psychological coherence, how assessments, curriculum, and teaching methods are aligned with how students feel, learn, and think. As AI systems mediate the learning experience, it is essential to ground their design and implementation in the established core principles of educational psychology: cognition and metacognition, learning, motivation, and beliefs about learning. These principles are the foundational pillars for understanding how students learn and adapt within AI-enhanced contexts. Considering study design, the earlier research has also often focused on the technical affordances of AI, the recognized possibilities for behavior that AI offers to a student or teacher, for example, that illustrate the relationship between AI's makeup (e.g., interface, etc.) and one's capabilities to use it (Wang et al., 2024). Technical affordances inform what a particular AI tool allows, inhibits, and welcomes, shaping how educational stakeholders utilize AI apps, platforms, and tools. These affordances such as accuracy (e.g., how reliable AI is, or how correctly a research method classifies or predicts; Hijji et al., 2023) or system architecture (i.e., model of the behavior, interactions, and structure of a system's parts, including data, hardware, and software to meet specific goals such as scalability; Dickerson and Mavris, 2010), however, do not appear to have sufficiently addressed the psychological mechanisms underlying learning outcomes.

As the field matures, the scholarly landscape has shifted from isolated empirical experiments toward a “synthetic layer” of research, where systematic reviews and meta-analyses seek to audit existing knowledge. However, a significant gap remains in aligning these rapid computational strides with robust psychological frameworks. Without this alignment, AI learning environments risk becoming inefficient delivery systems that fail to foster deep comprehension, equity, or lifelong learning skills. A comprehensive synthesis is required to map how the field is theoretically evolving and to determine if current research meaningfully integrates core foundational psychological principles into AI-mediated instruction.

## 2 Objectives

This study provides a systematic bibliometric synthesis of peer-reviewed scholarship published between 2016 and 2025 to evaluate the intersection of AI learning environments and educational psychology. The review follows a dual-pronged approach, examining both a synthetic corpus of 34 high-level review articles and a primary empirical corpus of 1,376 original research papers. We propose four specific objectives for this review. First, mapping the temporal and disciplinary evolution of AI in education to identify key inflection points in research volume and the transition from traditional adaptive systems to generative intelligence. Second, analyzing the operationalization of psychological constructs, specifically motivation, metacognition, self-regulated learning, and cognition, within AI-driven learning architectures. Third, evaluating the methodological ecology of the field to track the shift from exploratory computational modeling to rigorous quantitative validation (experimental and survey designs). Last but not least, identifying systemic gaps and ethical challenges in the current literature to focus on how AI-driven personalization addresses individual differences, equity, and the affective dimensions of human-AI interaction. By synthesizing this vast evidence base, the study aims to provide a roadmap for education stakeholders and researchers to leverage AI not merely as a technological tool, but as a psychological catalyst for enhanced educational outcomes.

## 3 Methods

To evaluate the intersection of AI learning environments and educational psychology, this study provides a systematic bibliometric synthesis of peer-reviewed scholarship published between 2016 and 2025. Following PRISMA 2020 guidelines, we employed a dualpronged approach, applying multiple descriptive and analytical modeling techniques to analyze a synthetic corpus of 34 systematic review papers and an empirical corpus of 1,376 original research articles. All screening workflows, inclusion/exclusion rules and analytical protocols are fully described; full replication is achievable using the documented database search strings and procedures.

### 3.1 Eligibility criteria

Following the PRISMA 2020 guidelines, study eligibility was determined through a multi-stage framework designed to align computational advancements with the foundational principles of educational psychology. The review period was restricted to peer-reviewed journal articles published between 2016 and 2025, a temporal window that captures the evolution from traditional adaptive learning systems to the contemporary rise of generative artificial intelligence and large language models (LLMs). To ensure a robust theoretical orientation, eligibility was contingent upon studies demonstrating an explicit empirical or theoretical grounding in core educational psychology constructs, specifically self-regulated learning (SRL), metacognition, cognitive load, motivation, and self-efficacy—as they are operationalized within AI-mediated learning environments.

The inclusion criteria were segmented to accommodate the study's dual focus on both the synthetic and empirical layers of research. For the synthetic corpus, eligibility was restricted to high-level evidence syntheses, including systematic reviews, meta-analyses, bibliometric studies, and scoping reviews. For the primary empirical corpus, inclusion required the presence of original data derived from quantitative, qualitative, and computational methodologies. All included studies were required to be published in English and situated within primary and secondary (K12) education, higher education, and general instructional settings to ensure a focus on the fundamental mechanisms of human learning rather than specialized professional training.

Conversely, a rigorous set of exclusion criteria was applied to maintain the study's focus on the intersection of AI architecture and psychological theory. Studies were excluded if their primary focus was situated within out-of-scope domains such as vocational training, business administration, higher education management, or digital entrepreneurship. Furthermore, purely technical engineering papers that detailed algorithmic development without examining pedagogical impact or human cognitive processes were omitted. Finally, clinical, medical, and therapeutic interventions were excluded unless they fundamentally addressed human cognition or beliefs about learning within an educational framework. We believe this selective distillation procedure ensured that the resulting corpus represented the most pedagogically relevant evidence at the nexus of technology and psychological science.

### 3.2 Information sources

A systematic search was conducted across six primary academic databases to ensure broad interdisciplinary coverage. These sources were selected to capture the intersection of computational science, pedagogical research, and educational psychology. The databases queried included: Social Sciences Citation Index (SSCI), Science Citation Index Expanded (SCIE), ERIC (Education Resources Information Center), Elsevier ScienceDirect, Springer Nature Journal Collection, and Web of Science (Core Collection). The search was restricted to peer-reviewed journal articles to maintain high standards of scientific rigor; conference proceedings, books, gray literature, and unpublished dissertations were excluded. The temporal window for all sources was set from January 1st, 2016, to December 31st, 2025, capturing the most recent decade of rapid transformation in AI learning environments. The final search was conducted in January 2026, and all retrieved records were exported as Research Information Systems (RIS) files to a centralized bibliometric pipeline for de-duplication and screening.

### 3.3 Search strategy

To facilitate a comprehensive audit of the field, a structured, multi-database search strategy was operationalized using a two-stage design. This bifurcated approach allowed for the simultaneous identification of the “synthetic layer” (secondary evidence syntheses) and the “empirical layer” (primary research). The search was executed across the six aforementioned repositories. The Social Sciences Citation Index (SSCI), the Science Citation Index Expanded (SCIE), ERIC, Elsevier ScienceDirect, the Springer Nature journal collection, and the Web of Science.

#### 3.3.1 Stage 1: synthetic corpus identification

The first search stage aimed to identify the “synthetic layer” of the field, focusing on state-of-the-art papers and evidence syntheses. Our search strategy relied on four conceptual pillars combined using Boolean operators (AND/OR): AI technology, educational context, psychological constructs, and methodological descriptors (Table 1). The first pillar identified specific AI technological architectures (e.g., “intelligent tutoring system*,” “large language model*,” and “LLM*”). The second pillar constrained results to educational contexts and pedagogical environments. The third pillar targeted core educational psychology constructs, including cognitive processes, metacognition, and epistemic beliefs. For this stage, we revised the methodological pillar to use review-specific terms, including “systematic review,” “scoping review,” “meta-analysis,” “mapping study,” and “synthesis.” This stage was critical for establishing the longitudinal trends and systemic gaps identified by the scholarly community when transitioning from data collection to broad scientific auditing.

**Table 1:** Pillars, search stage, and full search strings.

Pillar	Stage	Keywords and search strings
Pillar 1: AI technology	Universal	(“artificial intelligence” OR AI OR “intelligent tutoring system*” OR “machine learning” OR “adaptive learning system*” OR “generative AI” OR “large language model*” OR LLM*)
Pillar 2: Educational context	Universal	(education* OR schooling OR “learning environment*” OR instruction* OR pedagog* OR “learning communit*”)
Pillar 3: Psychological constructs	Universal	(cognition OR metacognition OR motivation OR “self-regulated learning” OR “epistemic belief*” OR “beliefs about learning” OR “cognitive process*” OR “individual difference*” OR “social interaction*” OR “learning theor*” OR constructivism OR behaviorism)
Pillar 4a: Review types	Synthetic corpus	(“systematic review” OR review OR “state of the art” OR “meta-analysis” OR bibliometric* OR “scoping review” OR “mapping study” OR “literature review” OR synthesis)
Pillar 4b: Empirical methods	Empirical corpus	(empirical OR quantitative OR qualitative OR “mixed method*” OR experiment* OR interview* OR questionnaire* OR “focus group*” OR “case stud*” OR ethnograph* OR “statistical analysis”)

#### 3.3.2 Stage 2: empirical corpus identification

The second search stage was designed to capture original empirical evidence while strictly excluding secondary research. Across both search stages, we kept the pillars for technology, context, and psychological constructs identical to ensure thematic alignment. For the methodological pillar, we used empirical descriptors (e.g., “experiment*”, “case stud*,” “mixed method*”) to ensure a focus on primary data. To maintain a strict empirical focus, the NOT operator was employed to exclude terms such as “review,” “meta-analysis,” and “bibliometric*.”

This dual-search design ensured that the final synthesized evidence base reflected both the granular experimental findings of the last decade and the high-level evaluative trends within the artificial intelligence in education (AIEd) community.

### 3.4 Selection process

To ensure the reliability and transparency of the corpus generation, the screening process was executed in two iterative stages corresponding to the synthetic and empirical layers of the investigation. Following the aggregation of records from the selected databases, Digital Object Identifier (DOI)-based deduplication protocols were applied to identify and remove redundant entries. Subsequently, the selection process diverged into two distinct pathways to isolate the respective evidentiary layers.

**Figure 1:**
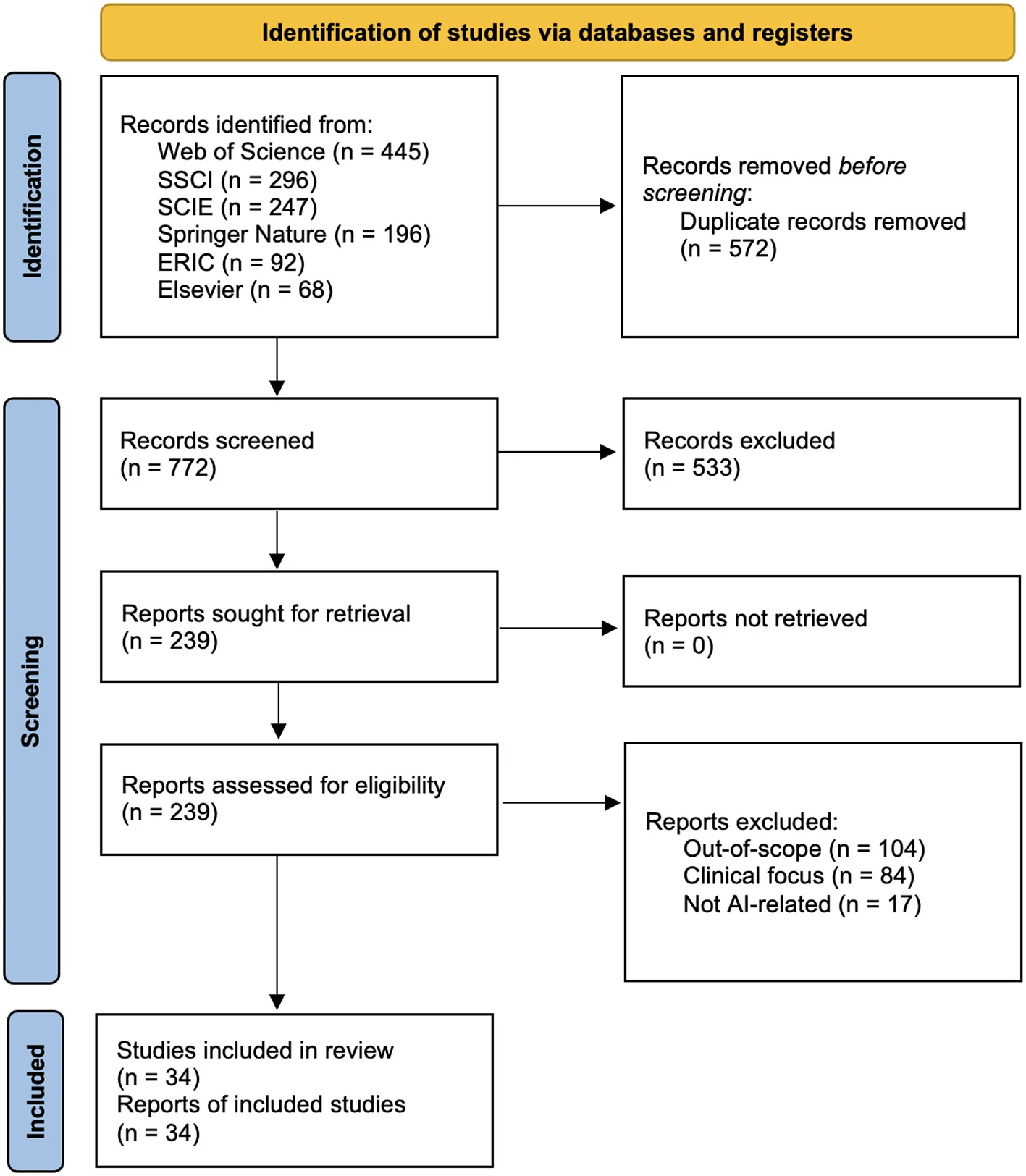
PRISMA flow diagram (synthetic).

For the synthetic layer, the initial search yielded a gross collection of records which, following deduplication, resulted in 772 unique articles. The first phase of screening involved a rigorous title and abstract review to isolate evidence syntheses, narrowing the corpus to 239 papers. These 239 papers were subjected to a full-text examination against the eligibility criteria, where studies focusing on clinical, medical, or strictly out-of-scope vocational fields were removed. Furthermore, a thematic filter was applied to ensure the literature was fundamentally anchored in the principles of educational psychology. This exhaustive distillation process refined the synthetic corpus to a final set of 34 highly relevant review articles, providing a robust overview of the field's synthetic layer (see Figure 1).

**Figure 2:**
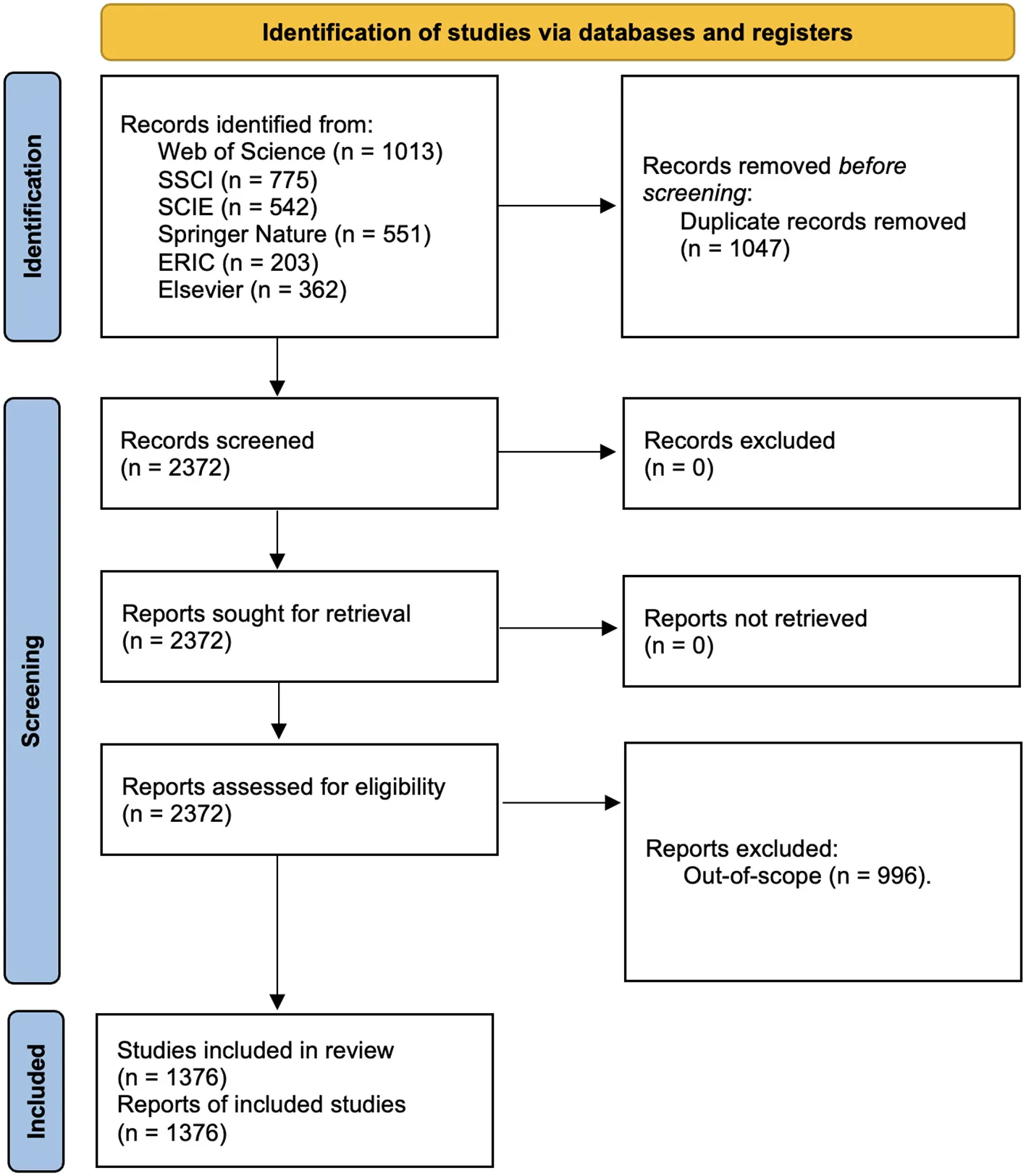
PRISMA flow diagram (empirical).

For the empirical layer, the search yielded a comprehensive raw collection which, after DOI-based deduplication, resulted in 2,372 unique empirical articles. These articles were then subjected to a multi-stage distillation process. First, active temporal and language filters were applied to ensure only English-language, peer-reviewed journal articles published between 2016 and 2025 were retained. Second, titles, abstracts, and author keywords were evaluated for explicit engagement with core psychological and cognitive constructs situated within a learning environment. Records with a primary focus on higher education administration, digital entrepreneurship, healthcare marketing, and technical engineering were omitted unless their findings fundamentally examined the psychological processes of the learner. This thematic refinement and rigorous multi-stage distillation resulted in a final corpus of 1,376 empirical records, establishing the primary evidence base for bibliometric and thematic analysis. The progression of this screening process is detailed in the PRISMA flow diagram (see Figure 2).

### 3.5 Data collection process and data items

A systematic extraction process was facilitated through a custom-developed R-based bibliometric pipeline. This automated workflow was designed to ingest and standardize Research Information Systems (RIS) files retrieved from disparate global repositories, which are ensuring consistency across heterogeneous metadata formats. To maintain data integrity and prevent the artificial inflation of publication metrics, a rigorous DOI-based deduplication protocol was strictly enforced. Records lacking Digital Object Identifiers were manually verified and treated as distinct entries only if they represented unique research contributions.

The variables extracted for analysis encompassed both standard bibliographic metadata and targeted indicators of evidentiary strength. For the entire corpus, the pipeline captured publication years, journal titles, author-defined keywords, and full abstracts. These data items served as the foundation for longitudinal volume analysis and lexical mapping. To clarify the disciplinary boundaries, metadata fields were normalized to ensure that variations in journal nomenclature and keyword phrasing did not bias the thematic synthesis.

For the empirical corpus specifically, the extraction process was augmented with advanced text-mining techniques to evaluate the scientific rigor of the primary data. Regular expressions (regex) were utilized to systematically identify and isolate psychometric indicators, including reliability coefficients (e.g., Cronbach's alpha) and reported performance indicators or effect sizes. This method allowed for a quantitative assessment of the “psychometric maturity” of the 1,376 empirical records, determining the extent to which AI-driven interventions were validated using established psychological standards. This automated feature extraction provided the necessary data to bridge the gap between technical AI performance and pedagogical efficacy. Rules for metadata standardization and deduplication are explicitly outlined in this section to support independent replication.

### 3.6 Study risk of bias assessment

To evaluate the internal validity and quality of the included literature, a dual-layered risk of bias assessment was implemented based on the criteria of “Psychometric Maturity” and “Theoretical Grounding.” For the synthetic layer consisting of 34 review articles, indicators of low bias were identified through standardized quality audits, and clear adherence to established reporting guidelines (e.g., PRISMA). For the empirical layer of 1,376 articles, the scientific maturity of the findings was assessed by analyzing the degree to which AI architectures were explicitly aligned with established educational psychology frameworks. This approach ensured that the synthesis prioritized studies with robust evidentiary strength over those characterized by purely technological novelty without pedagogical validation.

### 3.7 Synthesis methods

The synthesis employed a multi-modal analytical framework to transform raw bibliographic data into a structured conceptual roadmap. This process involved four distinct analytical streams:

1. **Bibliometric mapping:** lexical frequency analysis and “Knowledge Structure & Co-occurrence Networks” were generated using degree centrality as a proxy for conceptual influence. This allowed for the identification of pivotal nodes that served as bridges between technical algorithmic architectures and pedagogical outcomes.
2. **Thematic modeling:** latent Dirichlet Allocation (LDA) was applied to the metadata of the 1,376-item corpus to map “Structural Paradigm Shifts.” This longitudinal analysis tracked the field's transition from traditional “System Architecture” and rule-based models toward the contemporary paradigm of “Generative Intelligence.”
3. **Trend analysis:** to assess scholarly influence and adoption, temporal citation trajectories and adoption curves were calculated. This analysis specifically compared the rise of Large Language Models (LLMs) and Generative AI against the historical baseline of traditional Machine Learning and Neural Networks.
4. **Methodological ecology:** each study was classified into a methodological taxonomy (Quantitative Survey, Experimental, Qualitative, or Computational Machine Learning). This categorization enabled a systematic assessment of the field's evolution from exploratory computational modeling toward rigorous causal validation through experimental trials and structural equation modeling.

### 3.8 Certainty assessment

The overall certainty of the body of evidence was evaluated by comparing the longitudinal expansion of research volume against the stability of psychological constructs. A “Psychological Construct Proportion” analysis was operationalized to determine whether the significant inflection point in publication volume (notably the 2025 surge) was accompanied by a corresponding diversification in psychological theory. By assessing whether researchers were exploring new psychological domains or merely replicating findings regarding established variables (such as motivation and engagement), this assessment clarified the conceptual depth of the field. This allowed for a determination of whether the evidence base represented a maturing scientific discipline or a technologically driven expansion that was theoretically fragmented.

## 4 Results: synthetic layer

### 4.1 Study selection (*N* = 34 review articles)

The synthetic layer was identified through a structured multi-database search across six repositories designed to capture the intersection of four conceptual pillars: AI technologies, educational contexts, psychological constructs, and review methodologies. After duplicate removal, the search yielded 772 unique records (Figure 1). Because the aim of this stage was to map the field's evidence syntheses, screening was limited to secondary sources, including systematic reviews, meta-analyses, and state-of-the-art papers. Title and abstract screening reduced the dataset to 239 articles, which were subsequently assessed through full-text review. Studies with a clinical or medical orientation were excluded (84 studies). A final thematic filter ensured alignment with educational psychology by removing purely vocational, administrative, or technical applications. This staged filtering process produced a final corpus of 34 highly relevant review articles (Table 2). Although the search covered publications from 2016 to 2025, all included studies were published between 2022 and 2025, reflecting the field's recent movement toward greater scientific maturity.

**Table 2:** Overview of included synthetic corpus (systematic reviews, meta-analyses, and scoping reviews, *N* = 34).

No.	References	Article title
1	Huang M. et al., 2025	Exploring the development of personalizing learning in Chinese higher education: a systematic review of cognitive evolution engine by AI
2	Tohanean et al., 2025	Embedding digital technologies (AI and ICT) into physical education: a systematic review of innovations, pedagogical impact, and challenges
3	Xiong and Teo, 2025	Unveiling the effects of GenAI on motivation of learning English as a second/foreign language: evidence from meta-analysis
4	Huang W. et al., 2025	Chatbots and student motivation: a scoping review
5	Huang and Liu, 2025	The impact of technology on foreign language anxiety: a systematic review of empirical studies from 2004 to 2024
6	Sanabria-Z et al., 2025	Longitudinal bibliometric analysis of artificial intelligence in education research (1960-2023)
7	Lin et al., 2025	Constructionism in K-12 AI literacy education: a systematic review of pedagogical designs, student outcomes, and learning mechanisms
8	Zhao et al., 2025	Does generative artificial intelligence improve students' higher-order thinking? A meta-analysis based on 29 experiments and quasi-experiments
9	Ekizer, 2025	Exploring the impact of artificial intelligence on English language teaching: a meta-analysis
10	Zhuang et al., 2025	The affordances of artificial intelligence (AI) and ethical considerations across the instruction cycle: a systematic review of AI in online higher education
11	Arseven and Bal, 2025	Critical literacy in artificial intelligence assisted writing instruction: a systematic review
12	Chu et al., 2025	Concept maps in technological contexts of higher education: a systematic review of selected SSCI publications
13	Ogut, 2025	Assessing the efficacy of the Pomodoro technique in enhancing anatomy lesson retention during study sessions: a scoping review
14	Lin and Tan, 2025	A systematic review of generative AI in K-12: mapping goals, activities, roles, and outcomes via the 3P model
15	Chen and Cheung, 2025	Effect of generative artificial intelligence on university students learning outcomes: a systematic review and meta-analysis
16	Yang, 2025	AI-supported L2 vocabulary acquisition—a systematic review from 2015 to 2023
17	Zhang et al., 2025	Emotional artificial intelligence in education: a systematic review and meta-analysis
18	Lan and Zhou, 2025	A qualitative systematic review on AI empowered self-regulated learning in higher education
19	Ren et al., 2025	A systematic review exploring AI's role in self-regulated learning within education contexts
20	Septiana et al., 2024	Emotion-related pedagogical agent: a systematic literature review
21	Chen et al., 2024	A systematic review of research on immersive technology-enhanced writing education: the current state and a research agenda
22	Gavhane and Pagare, 2024	Artificial intelligence for education and its emphasis on assessment and adversity quotient: a review
23	Lin et al., 2024	A systematic review of big data driven education evaluation
24	Xia et al., 2024	A scoping review on how generative artificial intelligence transforms assessment in higher education
25	Barbosa et al., 2024	Adaptive learning in computer science education: a scoping review
26	Zhan et al., 2024	A systematic literature review of game-based learning in artificial intelligence education
27	Lo et al., 2024	Exploring the application of ChatGPT in ESL/EFL education and related research issues: a systematic review of empirical studies
28	Zhai et al., 2024	A systematic review of stimulated recall (SR) in educational research from 2012 to 2022
29	Halkiopoulos and Gkintoni, 2024	Leveraging AI in e-learning: personalized learning and adaptive assessment through cognitive neuropsychology—a systematic analysis
30	Deng and Yu, 2023	A meta-analysis and systematic review of the effect of chatbot technology use in sustainable education
31	Fang et al., 2023	A systematic review of artificial intelligence technologies used for story writing
32	Lee et al., 2022	Impacts of an AI-based chatbot on college students' after-class review, academic performance, self-efficacy, learning attitude, and motivation
33	Bennani et al., 2022	Adaptive gamification in e-learning: a literature review and future challenges
34	Sikström et al., 2022	How pedagogical agents communicate with students: a two-phase systematic review

All entries correspond to peer-reviewed journal articles included in the synthetic corpus (2022–2025). Article types follow standard systematic review typologies per PRISMA guidelines.

### 4.2 Study characteristics and lexical centrality

**Figure 3:**
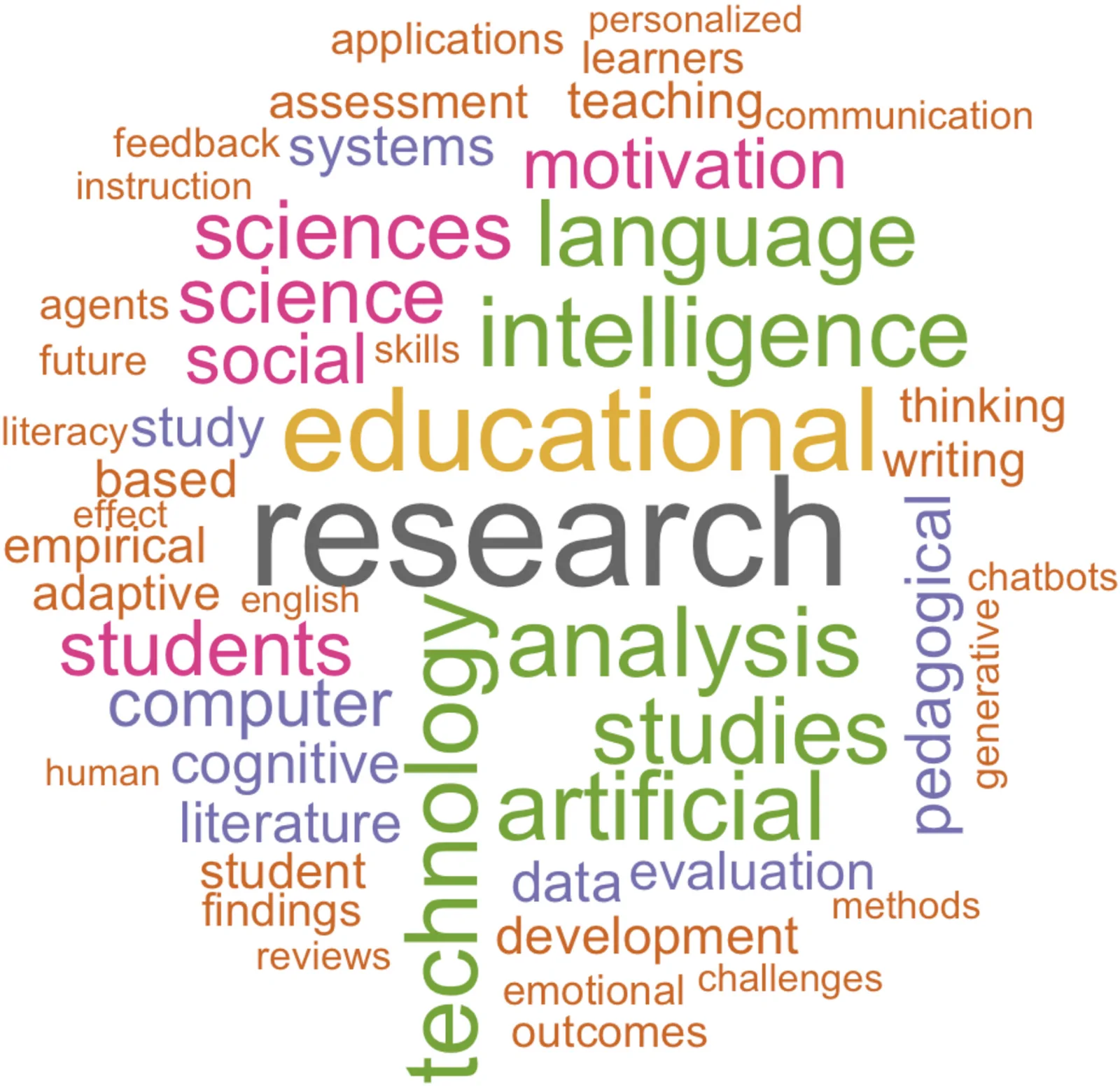
World cloud of the synthetic layer.

**Figure 4:**
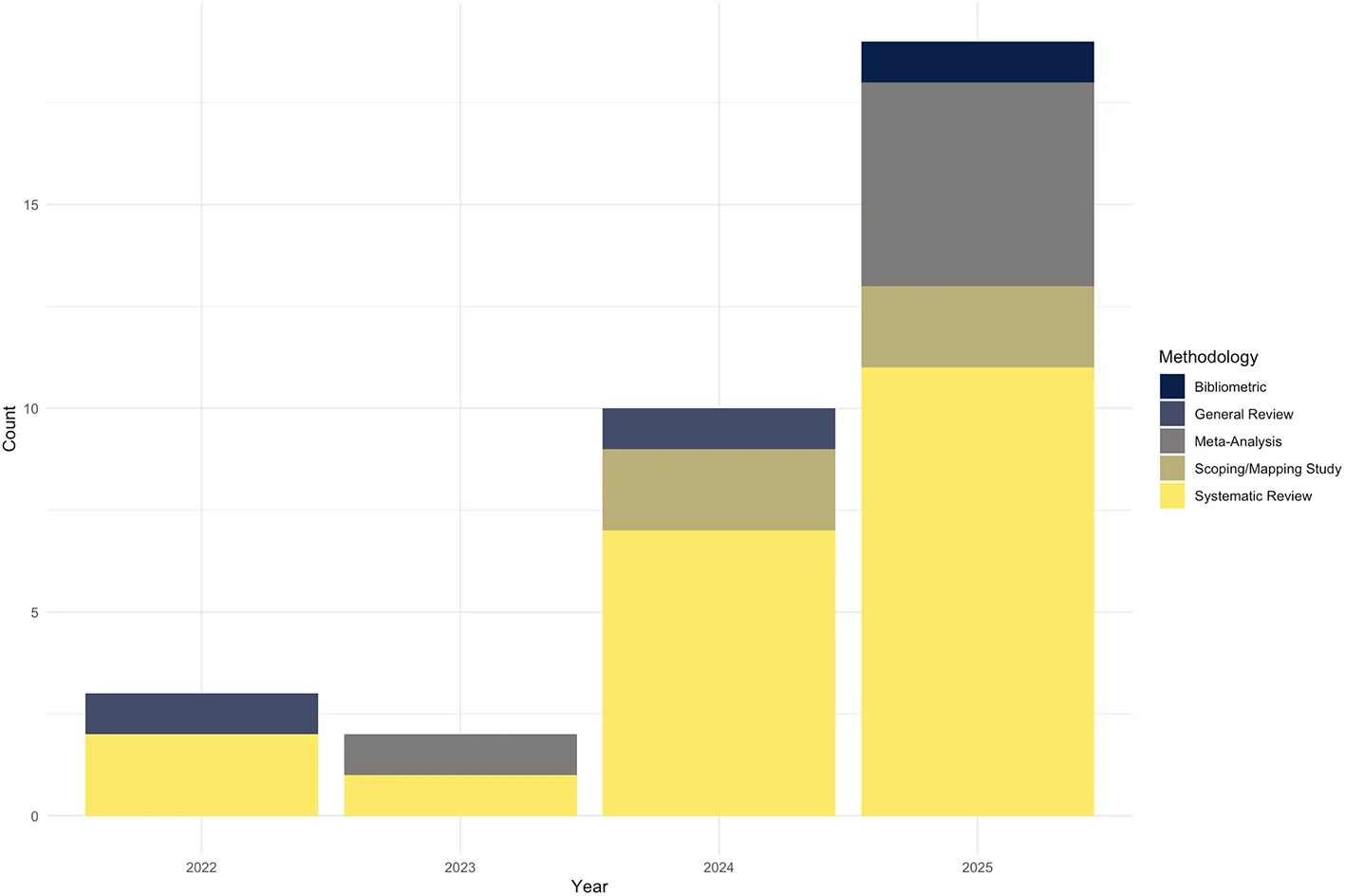
Primary focus of synthesized research.

The conceptual landscape of the synthesized literature is characterized by high thematic convergence. Lexical frequency analysis, as visualized in the Word Cloud (Figure 3), identifies “research,” “technology,” and “educational” as the most frequent descriptors, anchoring the corpus at the intersection of pedagogical science and digital innovation. Quantitative analysis, detailed in the Primary Focus of Synthesized Research (Figure 4), reveals that “language” and “social sciences” are the most prominent disciplinary domains, appearing in nearly 80% of the corpus. Crucially, the high frequency of psychological constructs such as “motivation,” “cognition,” and “pedagogical analysis” confirms that the current synthetic layer has moved beyond technical descriptions of AI, focusing instead on the human-centric variables that determine instructional efficacy.

### 4.3 Results of syntheses: temporal and historical trends

**Figure 5:**
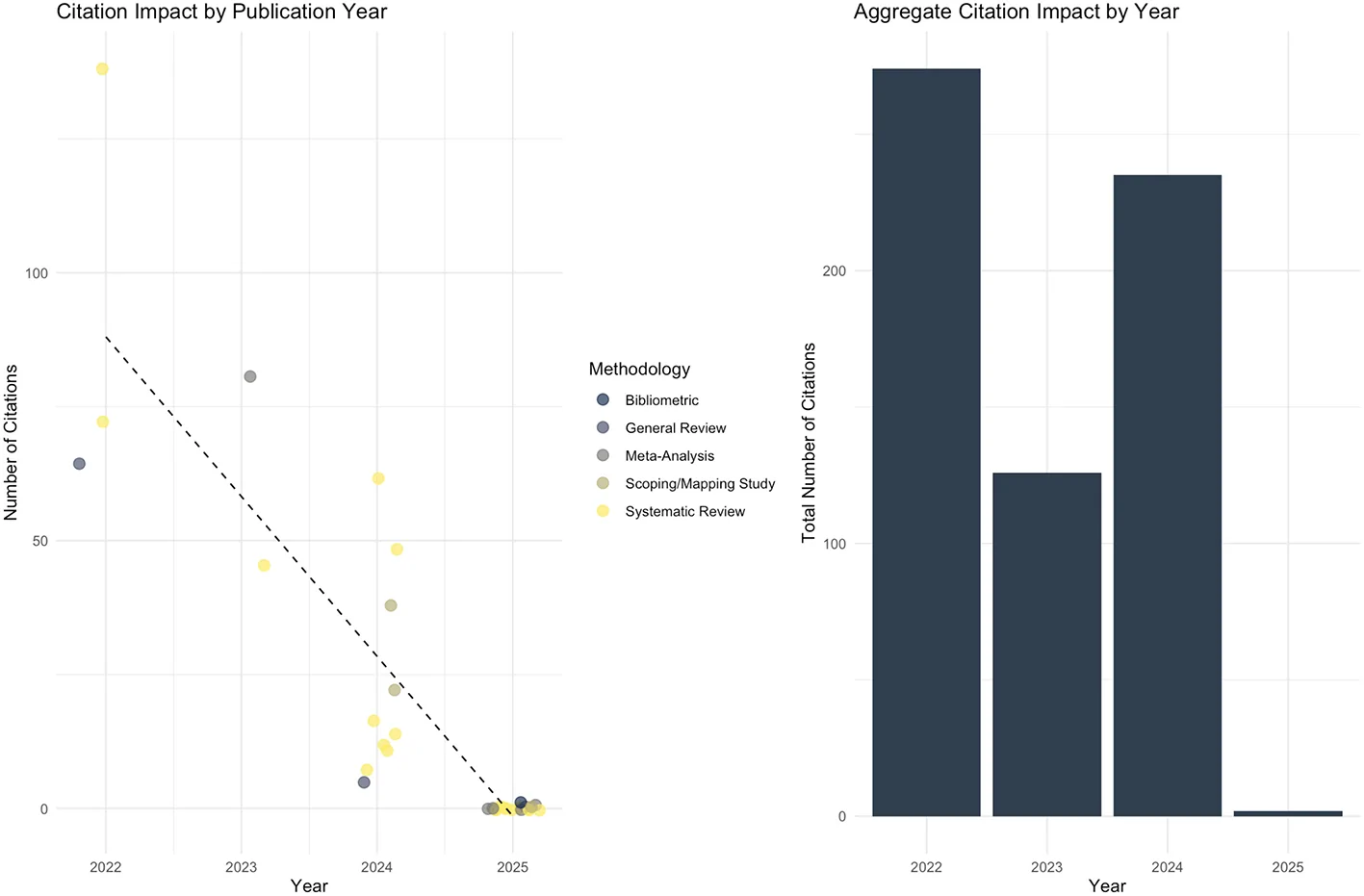
Citation impact by publication year.

An analysis of citation trajectories reveals the substantial impact of recent AI syntheses on the scientific community. As illustrated in Citation Impact by Publication Year (Figure 5), 2022 and 2024 represent significant “impact peaks.” The 2022 peak (approx. 280 aggregate citations) suggests that post-pandemic reviews established the foundational evidence base for the field. The 2024 peak likely reflects the rapid scholarly response to generative AI, where systematic reviews (highlighted in yellow) and meta-analyses (gray) became primary vehicles for knowledge dissemination.

**Figure 6:**
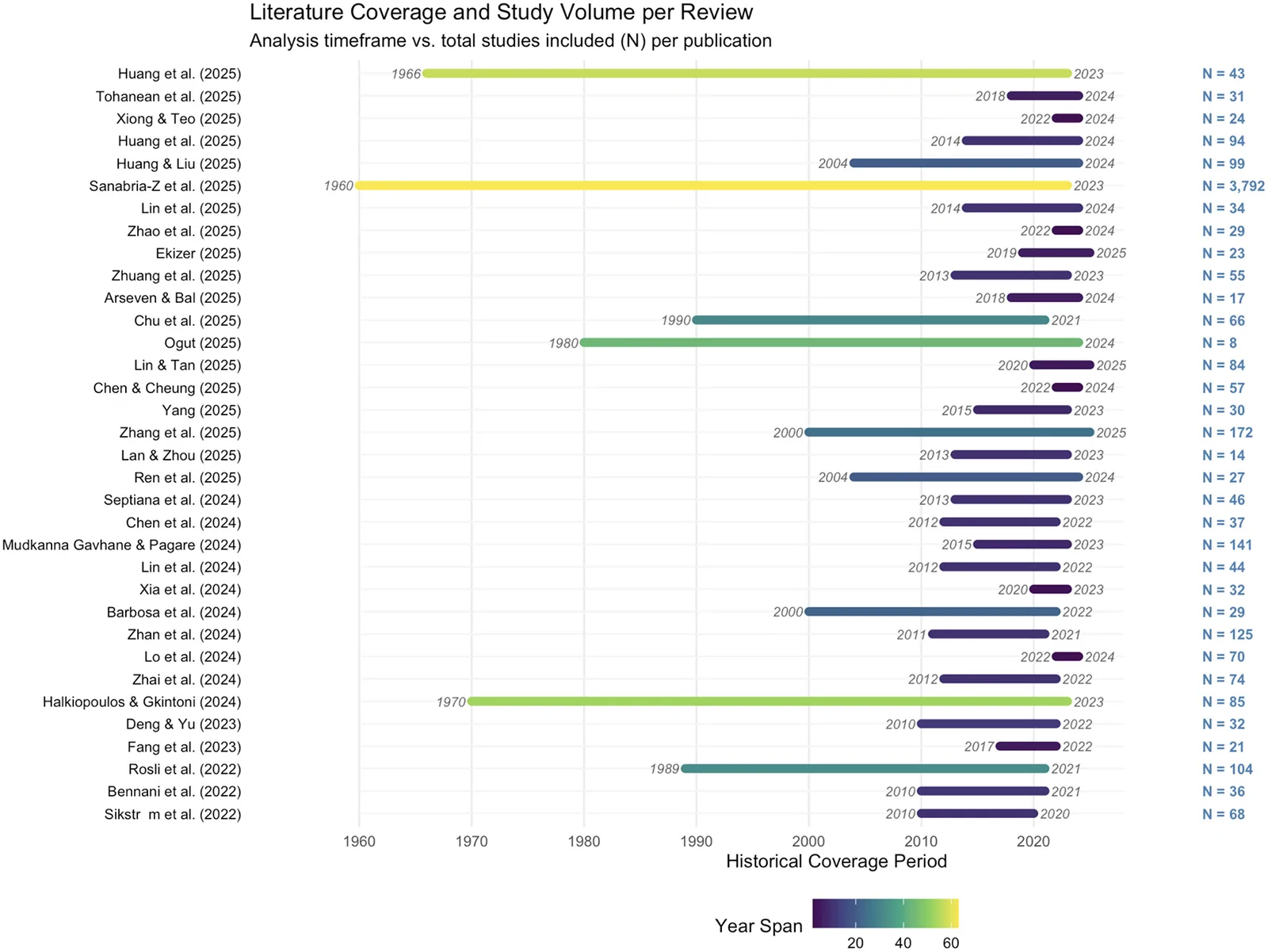
Literature coverage and study volume.

The longitudinal reach of the selected reviews demonstrates a field that is both deeply rooted and rapidly expanding. Figure 6 (Literature Coverage and Study Volume) highlights a balance between “modern era” snapshots and exhaustive historical audits. Specifically, (Sanabria-Z et al., 2025) represents a significant outlier, covering a historical span from 1960 to 2023 with a sample size of *N* = 3,792 studies. This juxtaposition ensures that contemporary AI applications are evaluated against both long-standing pedagogical theories and the latest technological breakthroughs, such as Large Language Models (LLMs) and immersive technologies. The typology of research has evolved from scoping reviews addressing targeted tools in 2022 (e.g., chatbots, pedagogical agents) to more diverse meta-analyses in 2023 and 2024 (e.g., story writing, immersive tech). The most dramatic expansion occurred in 2025, signaling a peak in scientific maturity characterized by a high volume of pooled effect sizes and standardized evaluations of how AI-driven environments foster effective instruction.

### 4.4 Geographic distribution of AI research in educational psychology

**Figure 7:**
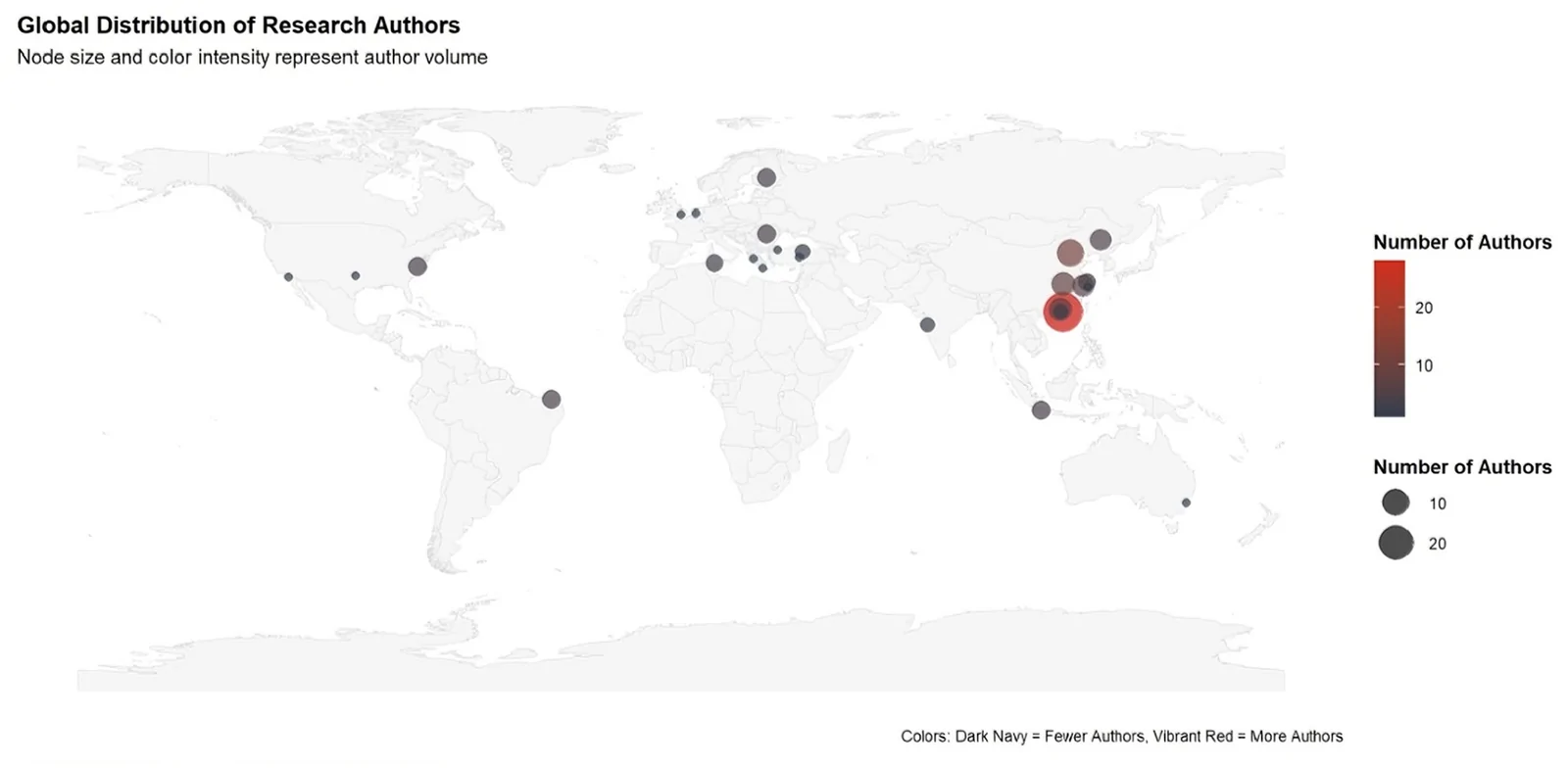
Geographic distribution of synthetic articles.

The geographic distribution of the identified synthetic articles underscores a concentrated global effort to audit the intersection of AI learning environments and educational psychology. The synthetic articles comprise 117 authors contributing to 34 peer-reviewed evidence syntheses, reflecting a high level of scholarly collaboration in the field (Figure 7). This collective effort represents a diverse global network dedicated to auditing the systemic intersection of artificial intelligence and educational psychology. East Asia stands out as the primary engine for high-density evidence synthesis, producing a significant portion of the systematic reviews, meta-analyses, and scoping reviews that define the field's current state-of-the-art. This intensity is mirrored by established research clusters in North America and across various regions of Europe, which together contribute heavily to the systemic mapping of technological and psychological pillars. The presence of authors from South America, Southern and Southeastern Asia, and Western Asia reflects a broadening international commitment to scientific auditing and the identification of research gaps. These patterns suggest that while the effort to establish longitudinal trends is geographically diverse, the global discourse on educational AI is currently most heavily shaped by scholarly hubs in East Asia.

### 4.5 Thematic synthesis: AI in pedagogical frameworks

The synthesis of the 34 articles provides a comprehensive roadmap organized around the following thematic pillars of educational psychology and AI.

#### 4.5.1 Typological distribution and temporal evolution

The longitudinal analysis of the identified literature reveals a significant escalation in the volume and complexity of evidence synthesis up to 2025. In the initial phase around 2022, research was represented by scoping and systematic reviews addressing targeted tools like chatbots, pedagogical agents, and adaptive gamification (Bennani et al., 2022; Lee et al., 2022; Sikström et al., 2022). The field matured significantly in 2023 and 2024, as the academic community produced a more diverse array of meta-analyses and scoping studies on specialized topics like story writing and immersive technology (Chen et al., 2024; Fang et al., 2023; Lo et al., 2024). The most dramatic expansion occurred in 2025, signaling a peak in the scientific maturity of artificial intelligence in education. During this year, the output of synthetic research grew exponentially across systematic reviews and meta-analyses (Chen and Cheung, 2025; Zhang et al., 2025). Additionally, the presence of various scoping and mapping studies suggests that the field is currently undergoing a process of rapid consolidation (Huang W. et al., 2025). This high volume of pooled effect sizes and quality audits demonstrates that the research community has moved beyond exploratory studies toward a standardized evaluation of how AI-driven environments foster effective instruction for all learners.

#### 4.5.2 The synthetic evolution of artificial intelligence in pedagogical frameworks

The landscape of modern education is undergoing a fundamental transformation characterized by the integration of AI to create more responsive and powerful learning environments. Analysis of recent literature indicates a significant shift from the generation of primary empirical data toward a robust synthetic layer of research driven by big data evaluation (Lin et al., 2024). By 2025, the field has reached a stage of scientific maturity where systematic reviews and meta-analyses have become the dominant modes of inquiry, moving beyond descriptive mapping toward auditing existing knowledge and pooling effect sizes to confirm AI's strong impacts on learning outcomes (Chen and Cheung, 2025; Zhao et al., 2025). These environments are no longer designed merely for content delivery but are structured to foster complex cognitive processes, including critical thinking, problem-solving, and metacognition across diverse disciplines such as computer science, medical education, and language acquisition (Barbosa et al., 2024; Ekizer, 2025; Ogut, 2025).

#### 4.5.3 Enhancing self-regulated learning and metacognitive development

A primary focus in current educational research is the role of AI in supporting self-regulated learning and metacognition (Lan and Zhou, 2025; Ren et al., 2025). Meta-analytical and review evidence highlights how AI-empowered systems provide students with the necessary tools to monitor, direct, and regulate their own learning processes. For instance, the implementation of automated corrective feedback and intelligent chatbots allows for the delivery of immediate responses that guide students through complex tasks and after-class reviews (Lee et al., 2022; Zhao et al., 2025). These AI-driven scaffolds help mitigate conceptual learning gaps, particularly in mixed-ability classrooms, by providing personalized interactive guidance and stimulated recall mechanisms (Zhai et al., 2024). Furthermore, the use of pedagogical agents based on communicative teaching models has shown promise in boosting self-efficacy, motivation, and socially shared regulation during collaborative learning activities, thereby enhancing individual and collective performance (Sikström et al., 2022).

#### 4.5.4 Personalization and adaptive instruction through intelligent systems

The drive toward inclusive and effective instruction for all learners has led to the development of sophisticated adaptive learning environments supported by cognitive neuropsychology and learning analytics (Halkiopoulos and Gkintoni, 2024). Modern AI systems now offer personalized assessment and tailored feedback that adjust to the specific needs of the learner, as seen with dynamic models like China's Cognitive Evolution Engine (Huang M. et al., 2025). In fields like computer science, these adaptive platforms identify struggling learners and provide targeted interventions based on learning styles to improve retention and achievement (Barbosa et al., 2024). The integration of gamification, serious games, and immersive technologies further enriches these environments, transforming meta-skills training by blending simulation with rigorous pedagogical goals (Bennani et al., 2022; Chen et al., 2024; Zhan et al., 2024). This personalized approach ensures that instruction remains relevant to the learners' prior knowledge, fostering a more equitable educational experience.

#### 4.5.5 Disciplinary applications and professional competency

The application of AI has proven particularly impactful in specialized academic and professional training, most notably in healthcare, physical education, and linguistics. GenAI is utilized to evaluate higher-order thinking and concept mapping in complex engineering and technology courses, allowing students to systematically build technical proficiency (Chu et al., 2025; Gavhane and Pagare, 2024). Similarly, in physical education, AI and ICT tools are employed to foster motor skill development, personalized real-time feedback, and educational inclusion, bridging the gap between technical assessment and human-centric coaching (Tohanean et al., 2025).

#### 4.5.6 Affective dimensions and human AI interaction

Beyond cognitive gains, the integration of AI into learning environments significantly influences the affective and social dimensions of education. Research into emotional AI and pedagogical agents reveals that the capability of AI to recognize and adapt to learners' emotional states can heavily impact student motivation, engagement, and anxiety (Septiana et al., 2024; Zhang et al., 2025). Recent reviews suggest that AI interventions can effectively reduce foreign language anxiety by providing psychological safety and personalized feedback, though designers must be careful to avoid inducing cognitive overload (Huang and Liu, 2025). The relationship between chatbots and motivation is generally positive, improving learning interest and engagement, yet sustained motivation often still requires elements of human interaction (Deng and Yu, 2023; Huang W. et al., 2025; Xiong and Teo, 2025). Educators must therefore balance the motivational benefits of AI chatbots and avatars with the need to maintain genuine human connection and social presence in digital environments.

#### 4.5.7 Ethical considerations and the future of AI literacy

As AI becomes a permanent fixture in the instruction cycle, addressing ethical implications and policy gaps remains a paramount concern for the academic community (Yan et al., 2025; Zhuang et al., 2025). Systematic reviews of AI ethics in education point to significant challenges regarding data privacy, algorithmic bias, and the potential for skill decay (use it or lose it phenomenon) or academic dishonesty if students become overly reliant on automated assistance (Lo et al., 2024; Xia et al., 2024). There is a growing emphasis on developing AI literacy and critical literacy among both students and teachers, utilizing constructionist pedagogies to ensure these tools are used responsibly rather than for direct copying (Arseven and Bal, 2025; Lin et al., 2025). This includes understanding the mechanisms of generative content, identifying the risks of hallucinations, and navigating the tensions between constructivist pedagogy and automated instruction in K12 settings (Lin and Tan, 2025). Moving forward, the objective is to create a synergy between human expertise and machine intelligence, ensuring that AI serves as a powerful catalyst for lifelong learning and professional excellence.

### 4.6 Certainty of evidence and ethical considerations

As AI becomes a permanent fixture in the instruction cycle, the certainty of evidence is increasingly tied to ethical integrity. Systematic reviews point to challenges regarding data privacy, algorithmic bias, and the potential for skill decay and academic dishonesty. Consequently, there is a growing emphasis on AI literacy and critical literacy among both students and teachers. Utilizing constructionist pedagogies ensures these tools are used responsibly, navigating the tensions between constructivist pedagogy and automated instruction. Moving forward, the scientific consensus suggests that the objective is to create a synergy between human expertise and machine intelligence, positioning AI not merely as a technological tool, but as a psychological catalyst to enhance educational outcomes and foster lifelong learning.

## 5 Results: empirical layer

### 5.1 Study selection (*N* = 1,376 primary studies)

The audit of primary empirical evidence was conducted through a multi-database Boolean search across SSCI, SCIE, ERIC, Elsevier ScienceDirect, and Springer Nature. After deduplication, the search produced 2,372 unique records. To ensure contemporary relevance, the dataset was limited to peer-reviewed English-language articles published between 2016 and 2025. A multi-stage screening process requiring explicit engagement with core psychological constructs (e.g., cognitive load, self-efficacy, and metacognition) refined the dataset to 1,376 relevant empirical studies. Articles focused solely on higher education administration or technical engineering without a psychological dimension were excluded to preserve alignment with the learning sciences framework.

### 5.2 Characteristics of primary studies: publication landscapes

**Figure 8:**
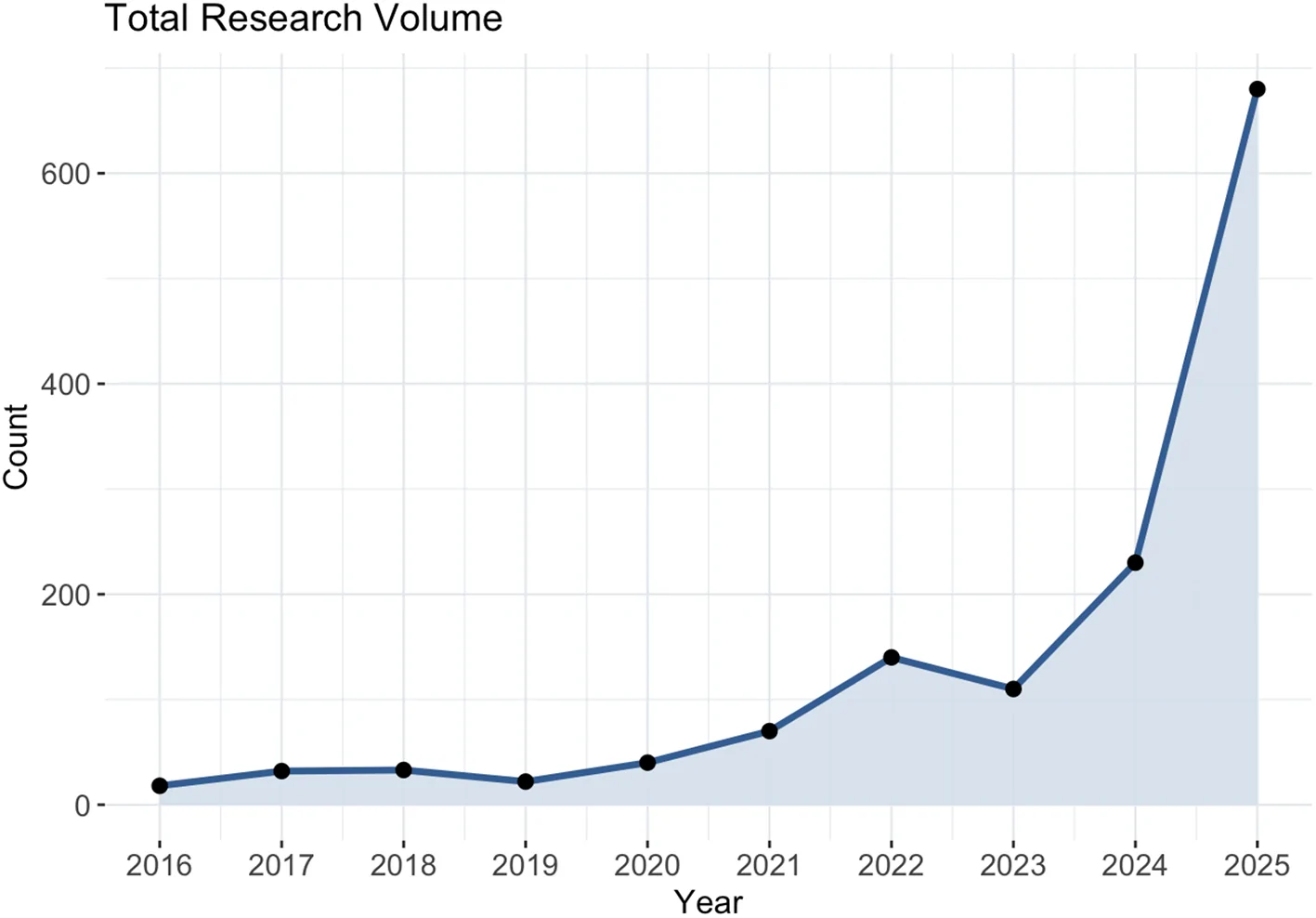
Total research volume.

**Figure 9:**
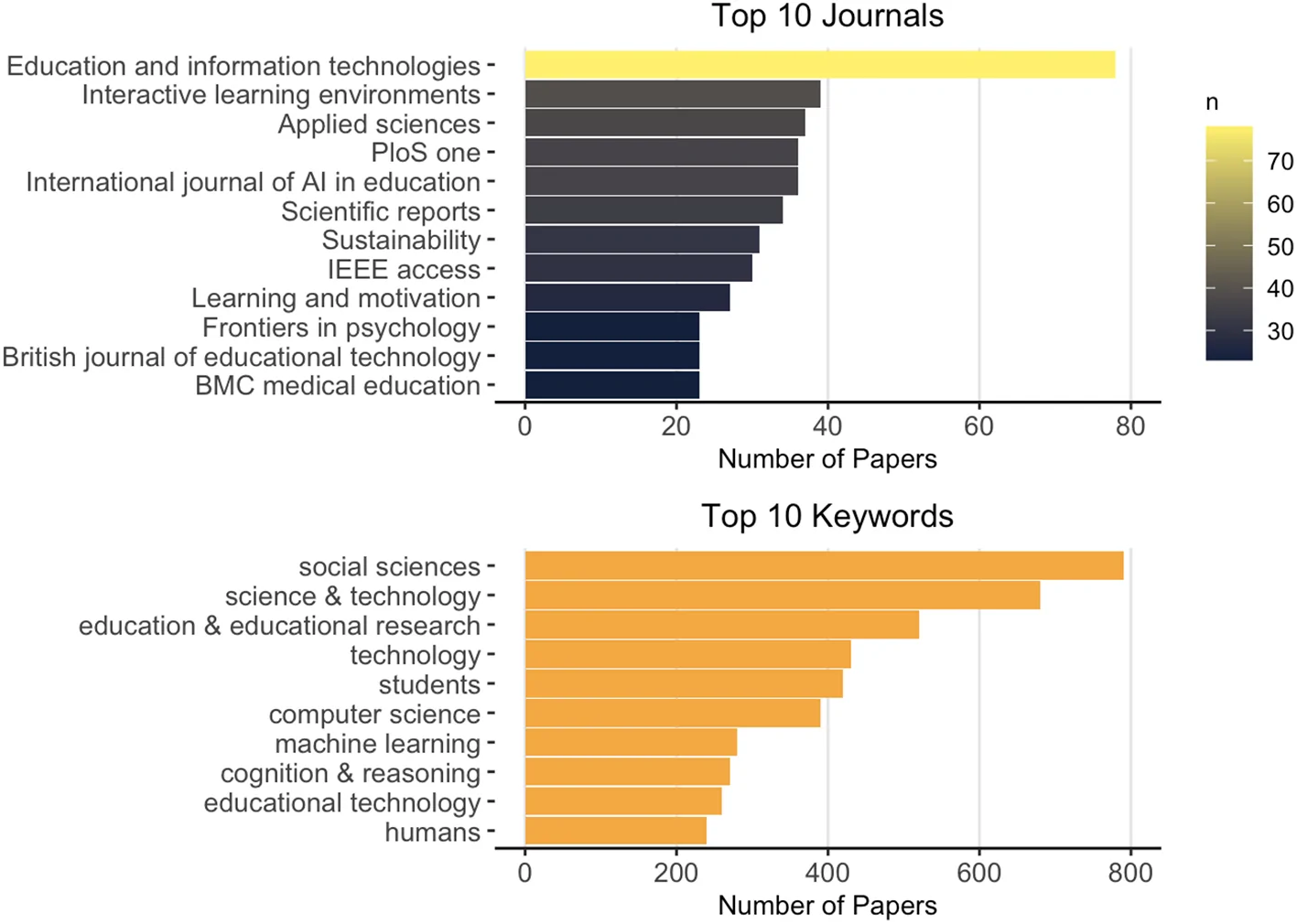
Top 10 journals and top 10 keywords.

The exponential growth of the field is most evident in the longitudinal distribution of research volume. As illustrated in Total Research Volume (Figure 8), the period between 2016 and 2020 was characterized by steady but modest output, averaging fewer than 50 papers annually. However, a significant inflection point occurred in 2021, culminating in an unprecedented surge to almost 700 empirical records by 2025. This rapid expansion is concentrated in high-impact venues; Top 10 Journals (Figure 9) identifies Education and Information Technologies as the premier dissemination channel, followed by Interactive Learning Environments and Applied Sciences. The presence of multidisciplinary journals such as PLoS One and Scientific Reports among the top outlets underscores the transition of AI-driven educational research into a globally recognized area of scientific inquiry.

### 5.3 Conceptual anchors and intellectual structure

**Figure 10:**
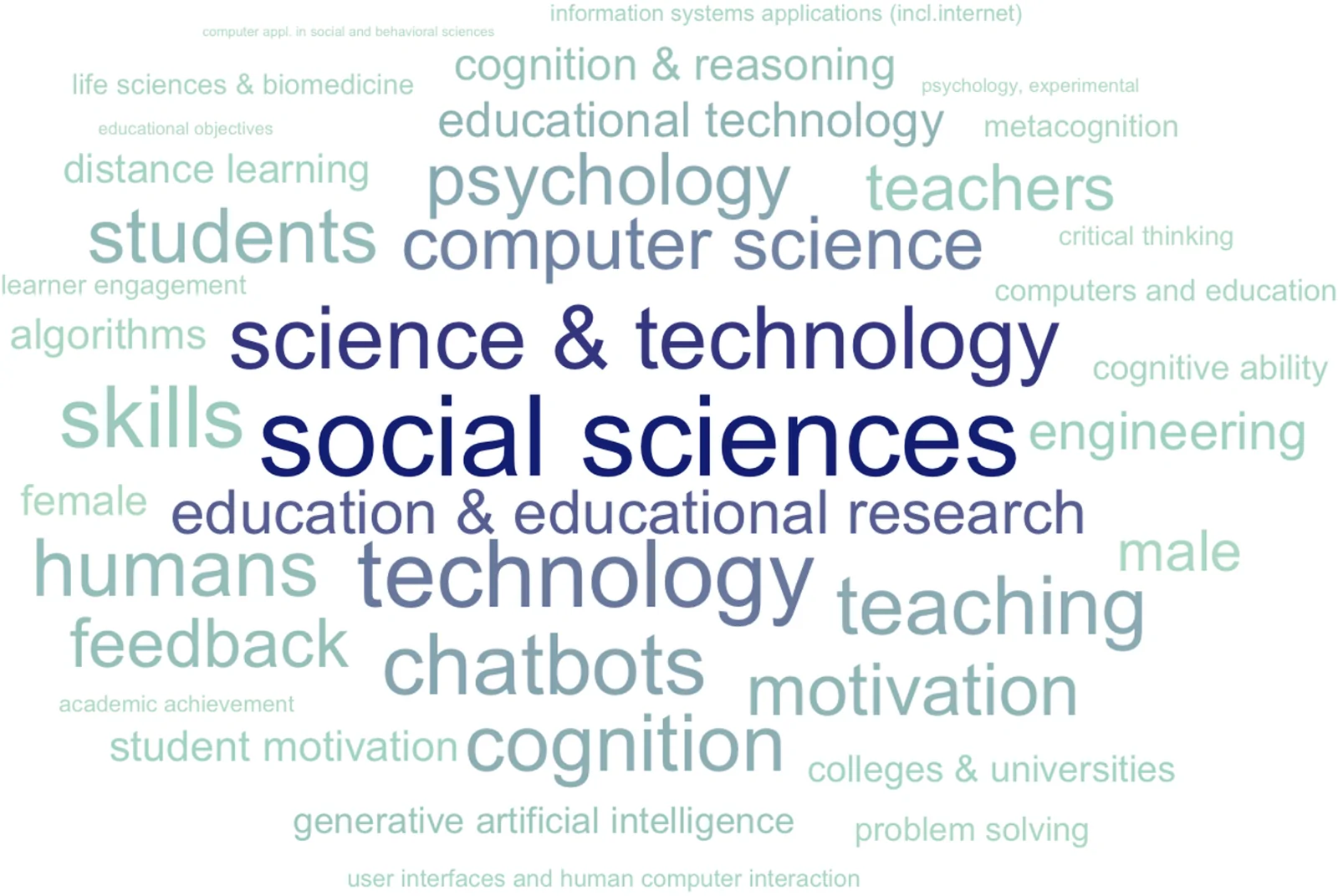
World cloud graphic from the empirical layer.

Bibliometric analysis reveals the disciplinary boundaries and intellectual connectivity of the field. Top 10 Keywords (Figure 9) and the Word Cloud (Figure 10) identify “Social Sciences” and “Science and Technology” as the two dominant pillars, appearing in nearly 800 and 700 records, respectively. This suggests a dual-focus research agenda that balances technical innovation with human-centric inquiry.

**Figure 11:**
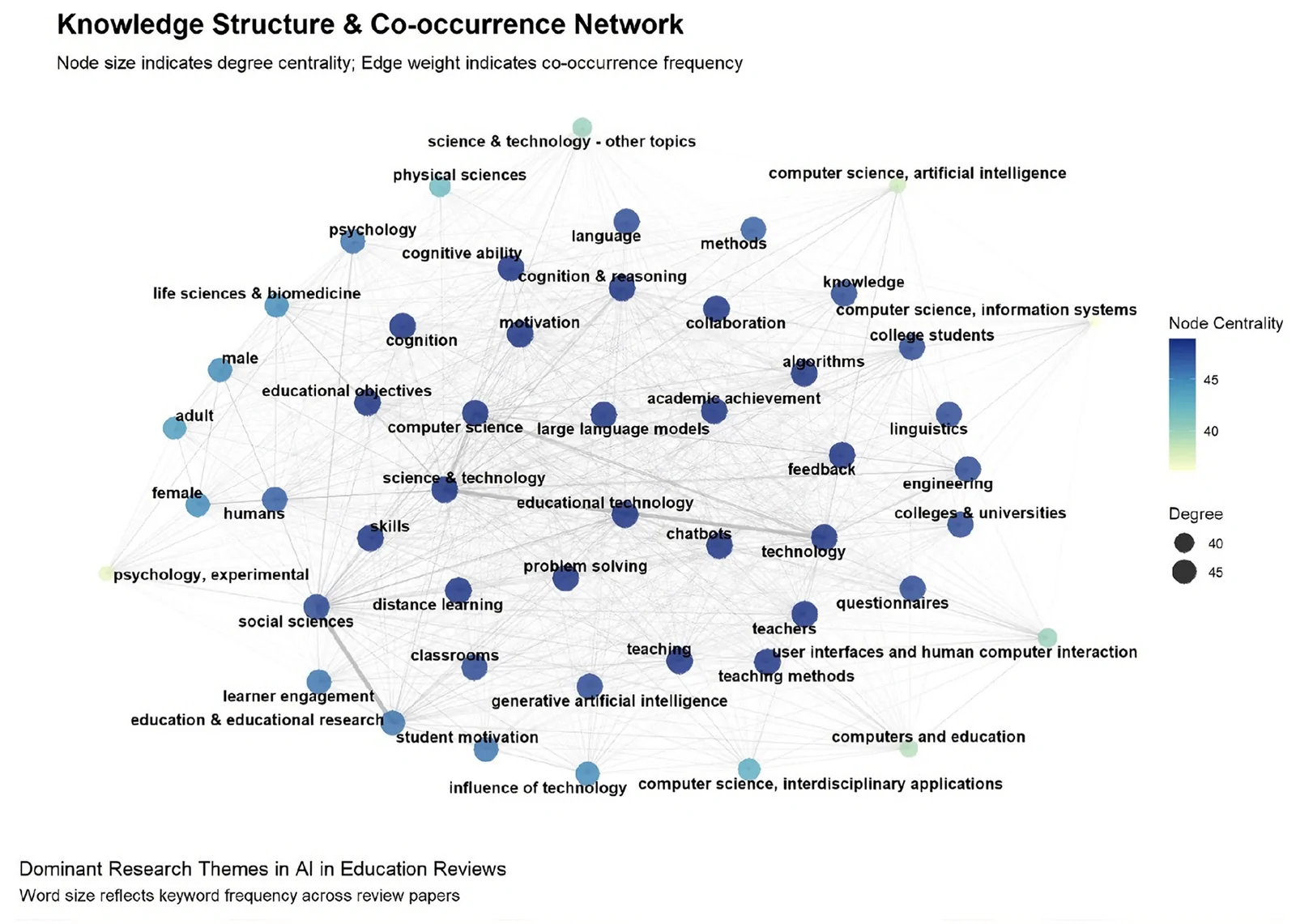
Knowledge structure and co-occurrence network.

The Knowledge Structure and Co-occurrence Network (Figure 11) demonstrates that psychological constructs, specifically “motivation” and “cognition,” serve as central nodes with high degree centrality, acting as conceptual bridges between technical domains (e.g., algorithms, machine learning) and pedagogical outcomes. Notably, the strong edge weights between “feedback,” “chatbots,” and “learner engagement” suggest a robust empirical cluster focused on utilizing conversational AI to mediate the affective dimensions of learning.

### 5.4 Technological and paradigm shifts

**Figure 12:**
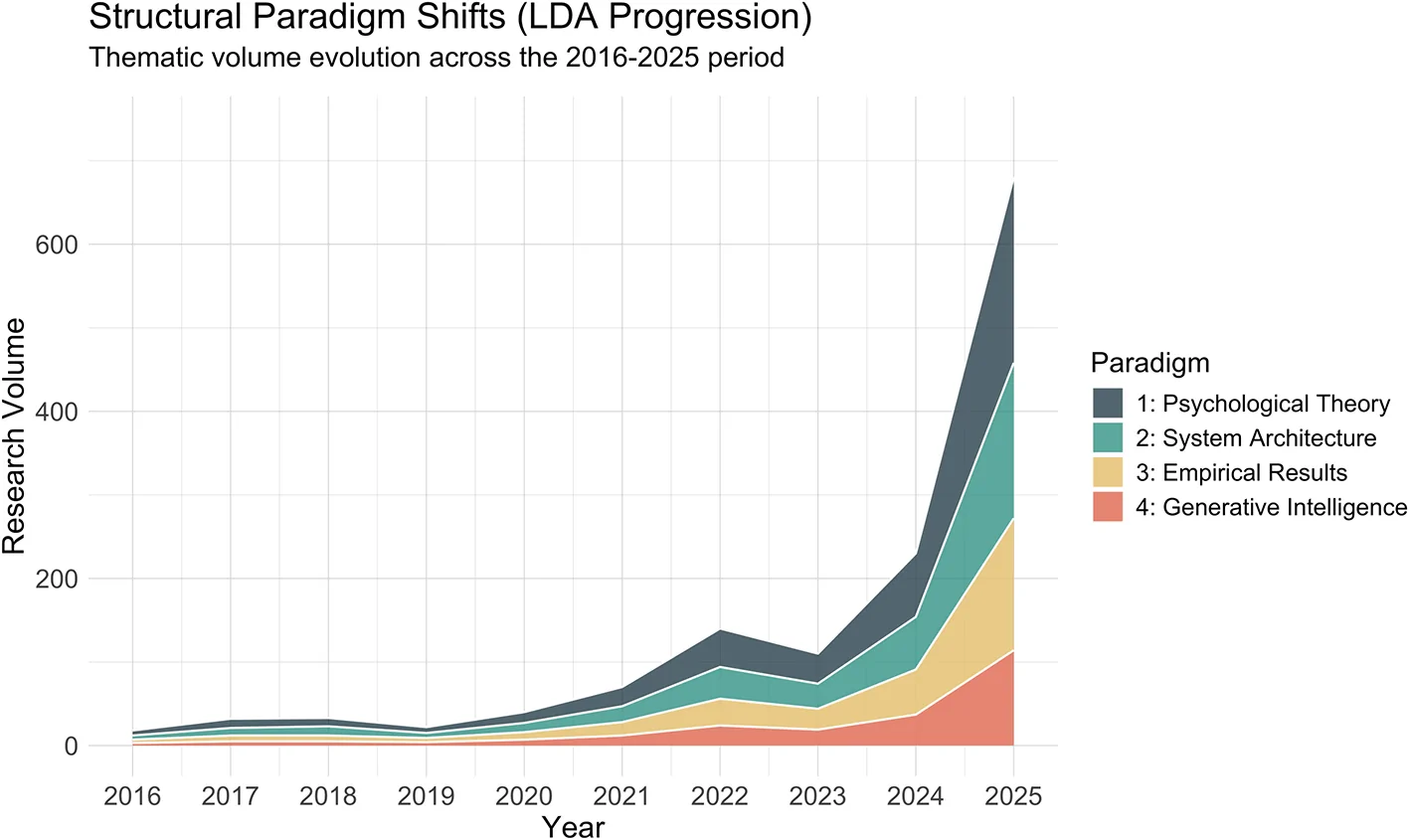
Structural paradigm shifts.

**Figure 13:**
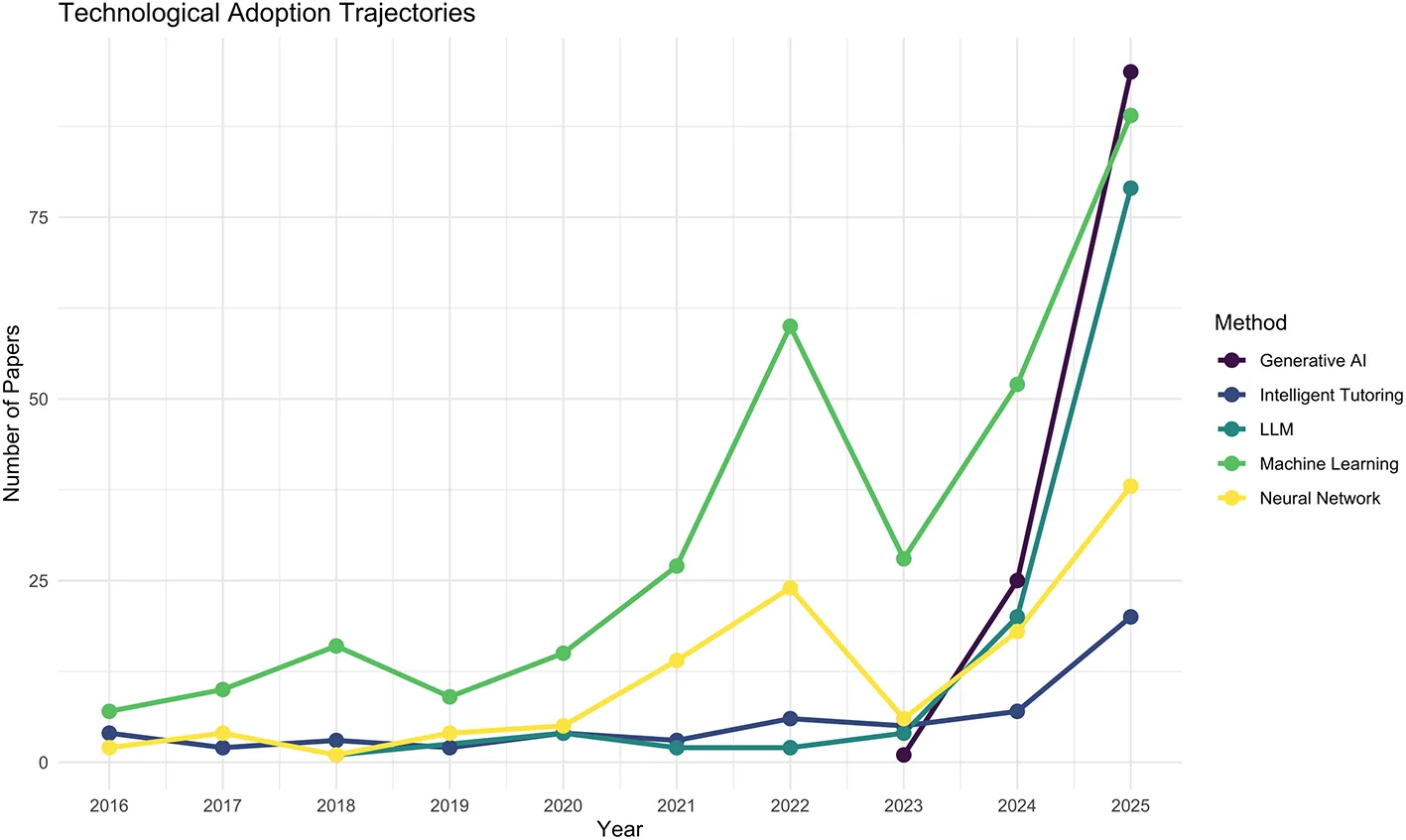
Technological adoption trajectories.

Thematic evolution, analyzed through Latent Dirichlet Allocation (LDA), reveals a shift in the field's structural focus. As shown in Structural Paradigm Shifts (Figure 12), while “Psychological Theory” remains the foundational anchor, there is a visible contraction in the “System Architecture” paradigm, suggesting that traditional, rule-based design is superseded by more adaptive models. The current volume peak is primarily driven by the “Generative Intelligence” paradigm, which has expanded exponentially since 2024. This trend is further clarified in Technological Adoption Trajectories (Figure 13), which tracks the transition from “Machine Learning” as the dominant empirical method (peaking in 2022) to the meteoric rise of “Generative AI” and “Large Language Models (LLMs)” between 2023 and 2025. These trajectories indicate a “generative turn,” where the research community has pivoted toward LLMs as the primary engine for pedagogical intervention.

### 5.5 Methodological ecology and psychological focus

**Figure 14:**
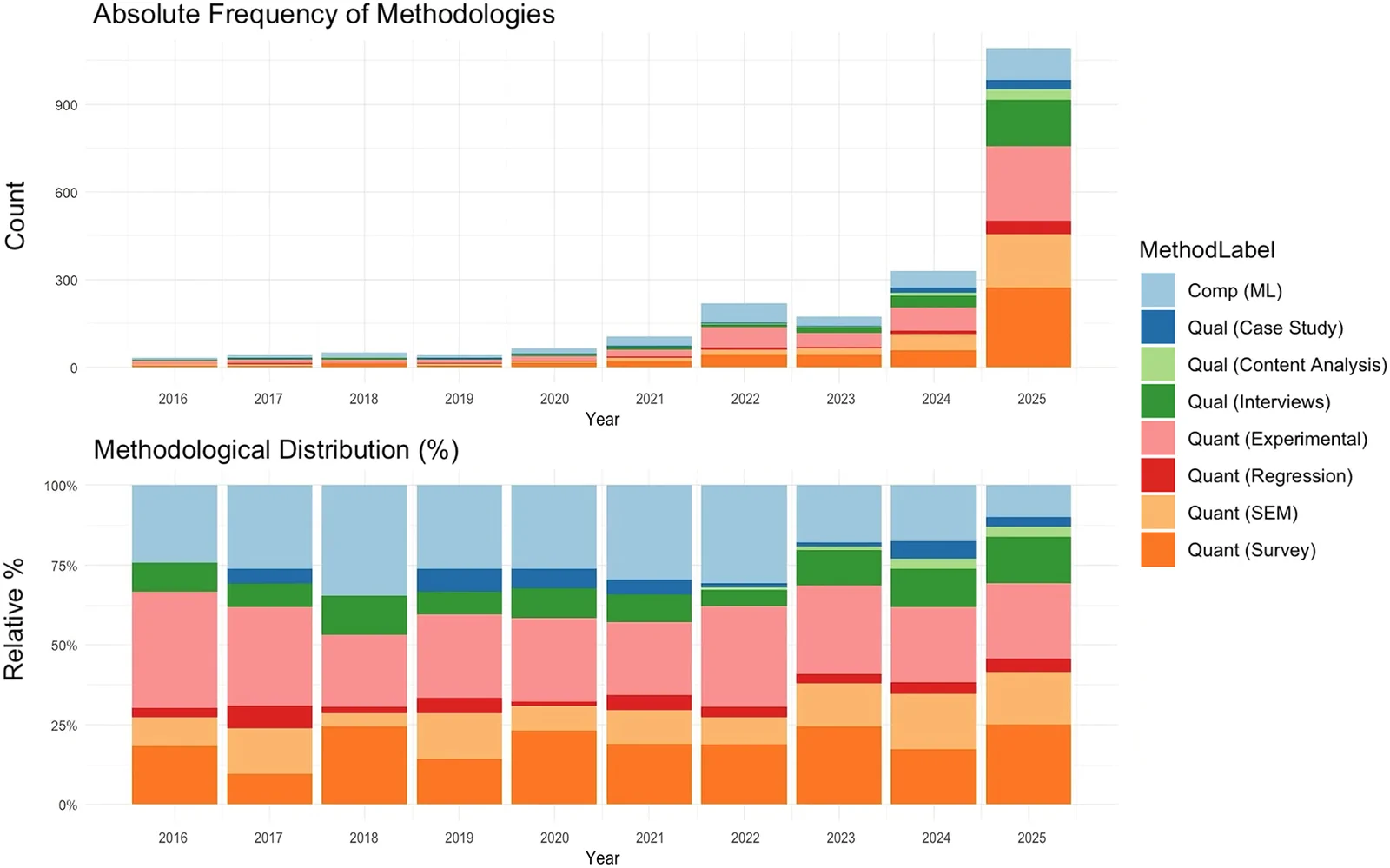
Absolute frequency of methodologies and methodological distribution.

The methodology of the field has undergone significant scaling in rigor. As visualized in Absolute Frequency of Methodologies and Methodological Distribution % (Figure 14), computational approaches (Comp ML) were prominent during the exploratory phase of 2016–2021. However, the current era is dominated by quantitative validation. By 2025, “Quant (Survey)” and “Quant (Experimental)” designs collectively represent more than 50% of the research volume. This shift toward experimental trials and structural equation modeling (SEM) indicates a maturing field focused on establishing causal links between AI interventions and learning gains.

**Figure 15:**
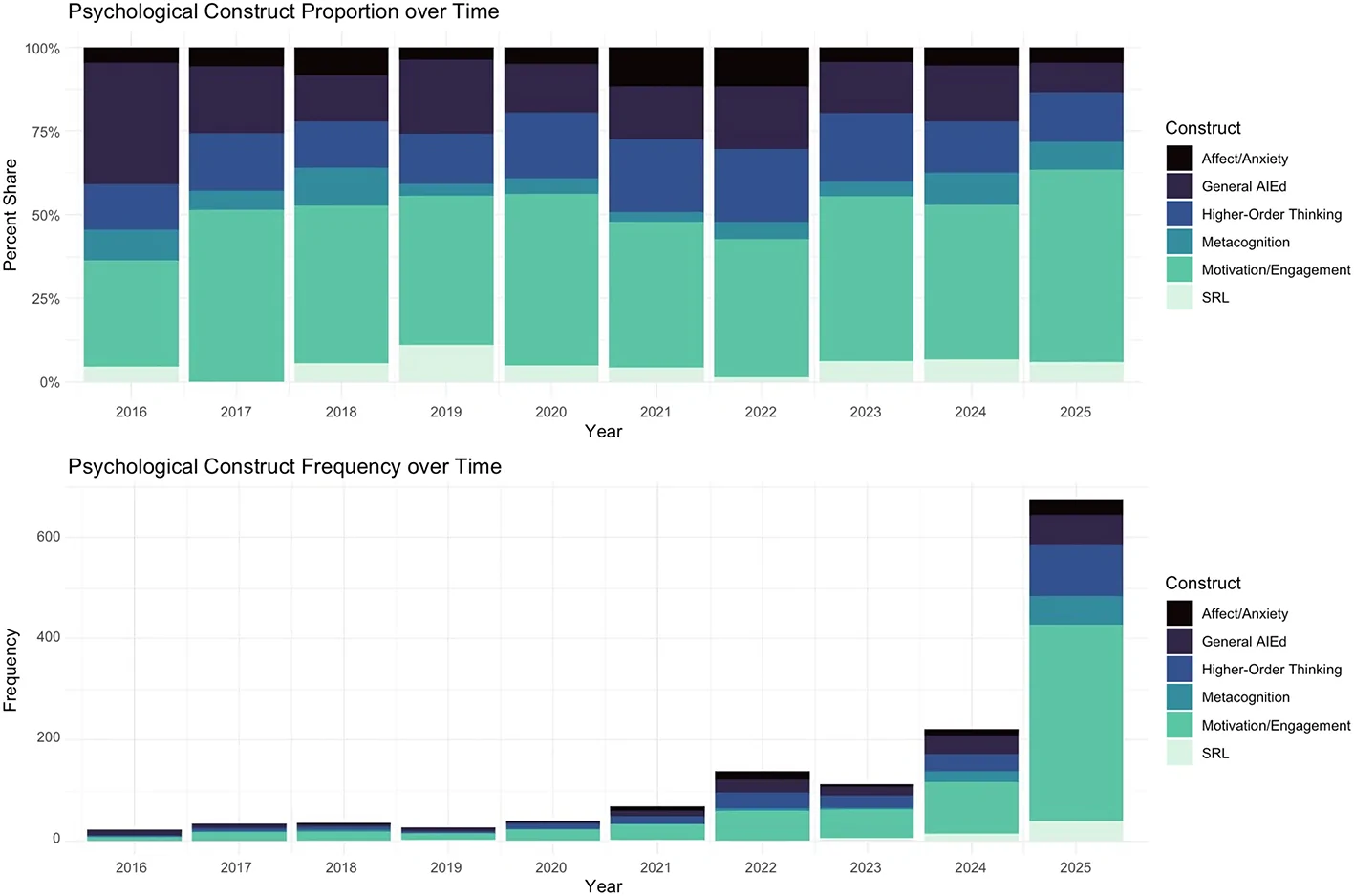
Psychological construct proportion and frequency.

In terms of construct operationalization, Psychological Construct Proportion and Frequency (Figure 15) reveals that “Motivation/Engagement” consistently holds the largest share of research attention. However, 2025 saw a massive absolute frequency spiking across all constructs, particularly “Higher-Order Thinking” and “Metacognition.” This suggests a move away from simple content delivery toward utilizing AI as a scaffold for complex, learner-centered regulation.

### 5.6 Theoretical gap

Despite the surge in empirical volume and methodological sophistication, a critical “theoretical gap” persists. While Figure 14 demonstrates an increase in rigorous quantitative reporting, Figure 15 indicates that the relative share of diverse psychological theories has remained static. This suggests that the field is technologically innovative but theoretically fragmented, often prioritizing successful technological outcomes (e.g., accuracy, retention) over the deep integration of foundational educational psychology principles. The evidence suggests that while the empirical community has successfully integrated conversational AI and LLMs, there remains a critical need for deeper alignment between AI system design and the established science of learning to move beyond technological novelty toward sustainable pedagogical coherence.

## 6 Discussion

### 6.1 Summary of evidence

The convergence of the synthetic (*N* = 34) and empirical (*N* = 1,376) layers reveals a field in the midst of an unprecedented “generative turn.” The longitudinal data indicates a modest output prior to 2020, followed by a significant inflection point in 2021 and a vertical surge peaking in 2025. This expansion is characterized by a definitive technological pivot: while Machine Learning served as the dominant empirical engine in the early 2020s, it has been rapidly superseded by Generative AI and Large Language Models (LLMs) as of 2024.

The evidence suggests that the research community has moved beyond technical exploration toward a “synthetic maturity,” where meta-analyses and systematic reviews now serve to audit pooled effect sizes. Central to this discourse is the role of AI as a “psychological catalyst” rather than a mere delivery tool. Constructs such as metacognition, self-regulated learning (SRL), and motivation have emerged as the primary conceptual anchors, with conversational AI (chatbots) serving as the most prevalent medium for facilitating real-time cognitive and affective support.

### 6.2 The interplay between synthetic consensus and empirical evidence

#### 6.2.1 Reciprocal validation and methodological maturation

A critical finding of this study is the reciprocal relationship between the “synthetic layer” and the “empirical layer.” The synthetic layer (*N* = 34) functions as a strategic compass, with the 2022 “impact peak” of reviews effectively predicting the methodological shift observed in the primary literature. While early reviews called for higher evidentiary standards to move beyond technological novelty, the empirical corpus (*N* = 1,376) demonstrates a delayed but robust response. This is evidenced by the transition in Figure 14, where the exploratory computational methods (Comp ML) dominant in 2016–2021 were superseded by the rigorous quantitative and experimental designs that peaked in 2025. This alignment suggests that the field's high-level self-evaluations (reviews) have successfully influenced the rigor of primary data collection, leading to a more mature and scientifically valid research ecology.

#### 6.2.2 Concurrent evolution in the generative era

The relationship between these two layers also reveals a unique temporal phenomenon regarding the “generative turn.” In most scientific disciplines, a “synthetic layer” of reviews typically lags several years behind empirical breakthroughs. However, our data reveals a concurrent evolution: the 2024 surge in high-impact systematic reviews on Generative AI (Figure 5) occurred almost simultaneously with the vertical spike in primary Generative AI and LLM studies (Figure 13). This suggests that the impact of large language models on educational psychology has been so profound that the scholarly community bypassed traditional lag times, engaging in real-time auditing and experimental validation in parallel. This synchronized growth underscores the urgency with which educational researchers are attempting to wrap psychological frameworks around rapidly advancing generative tools.

#### 6.2.3 The persistence of the rhetoric-reality gap

Despite the methodological alignment between the two layers, a “theoretical gap” remains a shared concern across the corpus. The synthetic reviews (e.g., Lan and Zhou, 2025; Zhang et al., 2025) consistently call for a more diverse integration of educational psychology principles beyond simple motivation. However, the empirical data reveals a stubborn “rhetoric-reality gap.” While the absolute frequency of studies has exploded (Figure 8), Figure 15 shows that the relative share of constructs such as metacognition and SRL has remained largely static over a decade. This reveals a critical tension: the synthetic layer has correctly identified the need for theoretical diversification, but the empirical layer—driven by the pressure of technological novelty—continues to prioritize a narrow set of established motivational outcomes. This suggests that while we are building more AI tools and auditing them more frequently, our theoretical “vocabulary” for interpreting their psychological impact has not yet expanded at the same rate as the technology itself. This observation is specific to the analyzed corpus and timeframe, and is not generalized as a universal epistemological trend across all AI education research.

### 6.3 Interpretation and context

The results demonstrate that AI learning environments are increasingly conceptualized as “psychologically situated systems.” The prominence of “Motivation/Engagement” in the empirical data aligns with the synthetic layer's focus on Emotional AI, suggesting that the field is successfully moving toward recognizing the affective dimensions of human-AI interaction. The rise of “Higher-Order Thinking” as a frequent construct in 2025 indicates a transition toward using AI to scaffold complex problem-solving and critical thinking, mirroring Vygotskian principles of the Zone of Proximal Development (ZPD; Smagorinsky, 2018).

However, a critical “theoretical gap” persists. Despite the shift toward rigorous quantitative methodologies, evidenced by the dominance of experimental trials and Structural Equation Modeling (SEM) in 2025, Figure 15 remains remarkably static. This suggests that while researchers are employing more sophisticated statistical tools, they are largely applying them to a narrow set of established variables. The field remains “theoretically fragmented,” often prioritizing technological outcomes (e.g., performance gains) over a deep, nuanced integration of foundational educational psychology principles. Computational innovation continues to outpace pedagogical coherence, creating a landscape where tools are built faster than their underlying psychological mechanisms are understood.

### 6.4 Strengths and limitations

The primary strength of this review is the multi-layered bibliometric approach, which cross-validated the “synthetic layer” of high-level scholarship against a massive “empirical layer” of 1,376 primary studies. The use of a custom R-based pipeline and LDA thematic modeling ensured a reproducible and objective analysis of paradigm shifts. However, several limitations must be noted. First, the rapid pace of the field means that studies published in late 2025 may not yet be fully reflected in citation impact metrics. Interpretations of bibliometric trends are constrained to this 2016–2025 corpus; broader cross-field epistemological generalizations are tempered by scope limitations. Second, the strict exclusion of vocational and administrative applications—while necessary to maintain the focus on educational psychology—may have omitted relevant cross-disciplinary insights into AI-driven skill acquisition. Finally, the reliance on English-language, peer-reviewed journals may introduce a geographical bias, potentially underrepresenting innovations from non-English speaking regions that are also leading in AI integration.

### 6.5 Implications and future directions

The synthesized findings of this review offer several significant implications for the diverse stakeholders within the artificial intelligence in education (AIEd) ecosystem. For researchers, there is an urgent mandate to transcend the “technological novelty” that has historically characterized much of the empirical surge in the field. As the research volume moves toward a state of scientific maturity, future inquiries must prioritize deeper theoretical alignment, shifting from the assessment of surface-level motivational measures toward investigating the profound ways in which AI alters learners' epistemic beliefs and long-term metacognitive habits. This evolution requires moving beyond the “does it work?” paradigm to a more nuanced exploration of the cognitive and psychological mechanisms that underpin human-AI collaborative learning.

Simultaneously, the “generative turn” documented in this study necessitates a fundamental shift in the design and deployment of AI architectures. Designers and developers must transition toward “human-centric architecture,” where the primary objective of a system extends beyond technical accuracy to encompass psychological safety and the optimization of cognitive load. In the current era of large language models (LLMs), it is imperative that these systems are engineered to function as pedagogical scaffolds rather than cognitive crutches. By ensuring that AI-driven interventions promote active engagement and deep comprehension rather than passive reliance, designers can help preserve the learner's agency and foster the development of higher-order thinking skills within AI-mediated environments.

From a policy and ethical standpoint, the unprecedented escalation in research volume and technological application demands the institutionalization of robust AI literacy and ethical frameworks. As generative tools become permanent fixtures in the instruction cycle, policymaking must proactively address the critical risks highlighted in the 2025 synthetic reviews, including algorithmic bias and the potential for cognitive skill decay. Stakeholders must advocate for a critical literacy approach, grounded in constructionist pedagogies, that empowers both educators and students to navigate the tensions between automated instruction and human-centric learning. This ensures that the integration of AI serves to promote educational equity and inclusive practice rather than exacerbating existing digital and cognitive divides.

To advance the field, researchers should develop consistent definitions and metrics to operationalize scientific maturity within AI education research. Evaluations of research quality must account for rigorous study designs, full reporting of psychometric properties, transparent methodological documentation, and reproducible analytical procedures (Chen and Cheung, 2025). Establishing unified standards for scientific maturity will support reliable cross-study comparisons and encourage widespread adoption of rigorous empirical practices across computational, experimental, and survey-based investigations of AI learning environments.

Explicit operationalization of psychological maturity represents another vital direction for future work. Within AI-mediated learning contexts, psychological maturity refers to the extent to which research fully engages with complex educational psychology constructs, rather than limiting assessments to superficial measures of motivation and engagement (Lan and Zhou, 2025). Formalized guidelines for this construct will help researchers evaluate how AI tools support advanced learning processes such as metacognition and self-regulated learning, moving beyond narrow focus on basic affective outcomes.

Scholars should also formalize clear criteria to define and assess the persistent theoretical gap identified in this synthesis. This gap describes a widespread disconnect between rapid advancements in AI technology and insufficient integration of established educational psychology theories into empirical research and system design (Zhang et al., 2025). A standardized definition will allow researchers to systematically track this disconnect over time and design studies that better ground AI development in foundational learning theories.

In conclusion, while AI has demonstrated immense potential as a psychological catalyst to enhance educational outcomes, the field has reached a critical juncture that necessitates a phase of rigorous theoretical consolidation. The transition from an era of exploratory technological innovation to one of standardized pedagogical evaluation marks a significant milestone in the maturity of the field. To foster truly effective, inclusive, and equitable learning environments, the next generation of AI research must ensure that computational progress is no longer an isolated endeavor but is instead firmly anchored in the foundational principles of the science of learning. Only through this deep alignment of machine intelligence and educational psychology can AI serve as a sustainable engine for lifelong learning and professional excellence for all.

## Data Availability

The datasets presented in this article are not readily available because data are extracted directly from public databases. Following the searching criteria described in this article, researchers will be able to acquire the exact datasets used for this study. Requests to access the datasets should be directed to shujin.zhong@unf.edu.
